# Supplementary material for: Targeted Removal of Galloylated Flavanols to Adjust Wine Astringency by Using Molecular Imprinting Technology
Source: Foods. 2023 Sep 5;12(18):3331. doi: 10.3390/foods12183331 (PMC10530250; doi:10.3390/foods12183331)
Supplement: Supplementary file 1 [file foods-12-03331-s001.zip › foods-2522112-supplementary.pdf]

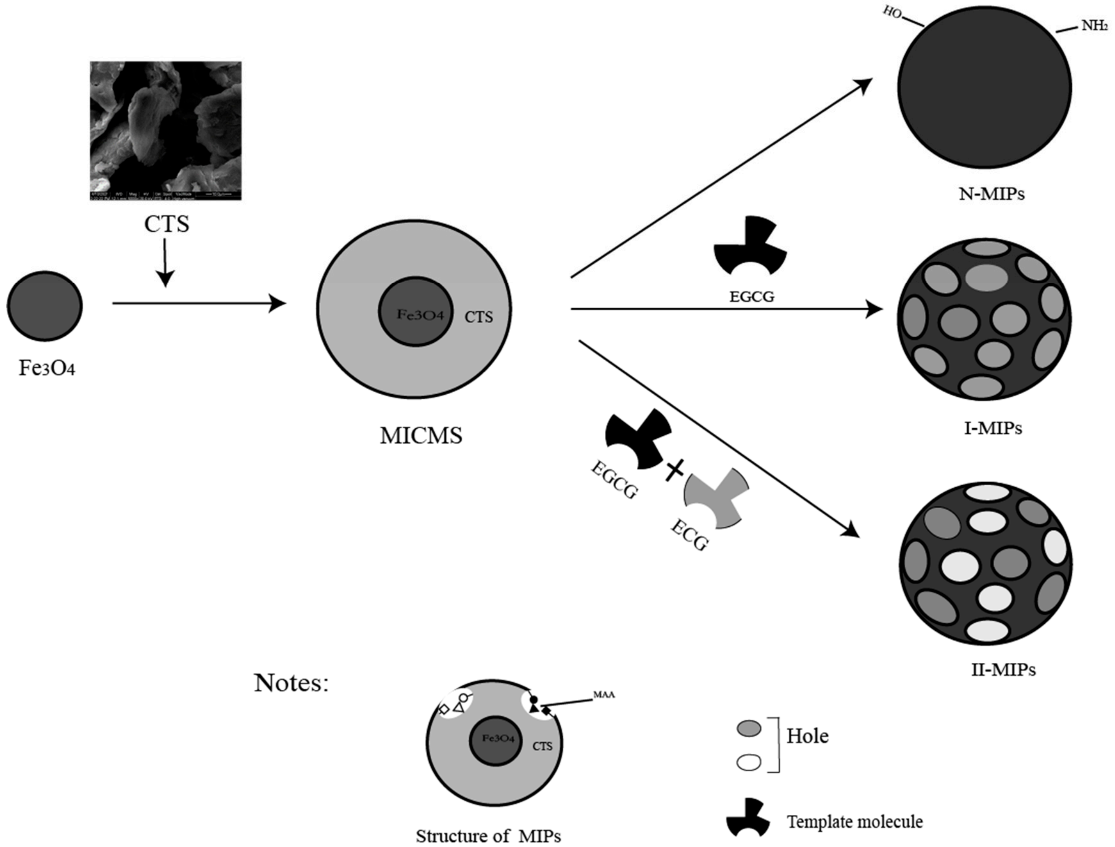

Figure S1. Molecular imprinted polymer (MIPs) material synthesis process model. The CTS (Chitosan) was wrapped on the surface of Fe_3O_4 to form Molecularly imprinted chitosan microspheres (MICMS). EGCG (Epigallocatechin gallate) and ECG (Epicatechin-3-O-gallate) were used as template molecules for MIPs. MAA (Methacrylic acid) was functional monomer. N-MIPs was MIPs without template molecule. Chitosan functional groups hydroxyl and amino groups are present on the surface of N-MIPs; I-MIPs was MIPs synthesized with EGCG as template molecule; II-MIPs was MIPs synthesized with EGCG and ECG (1:1, mol/mol) as template molecule.

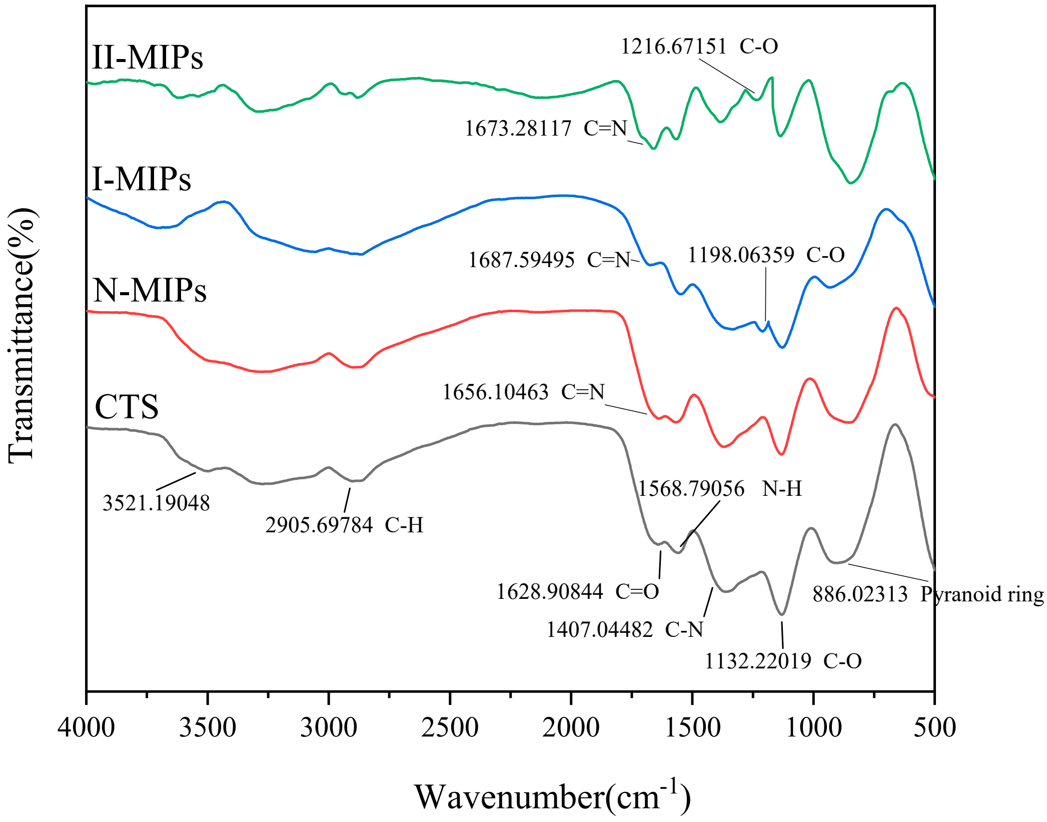

Figure S2. Fourier infrared spectra of MIPs (N-MIPs, I-MIPs, II-MIPs) and CTS.

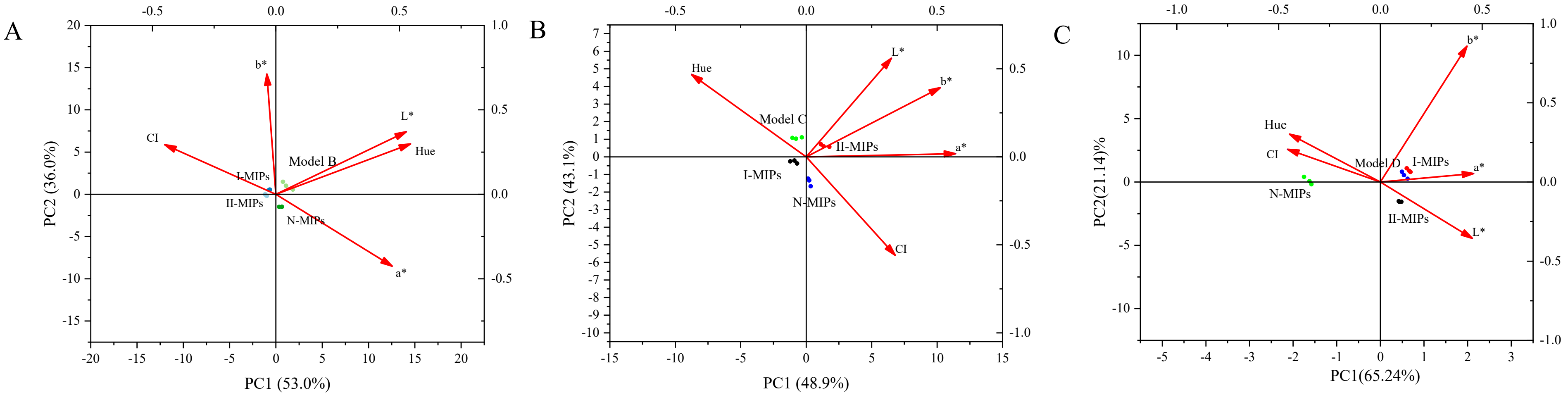

Figure S3. Effect of MIPs on color parameters of model solutions. B/C/D correspond to the green tea model solution, grape seed model solution and grape skin model solution, respectively. a*, b*, L*, CI and Hue are the color space parameter. The colour space of Model A was not explored in this work, due to the small amount of colour-presenting substance in Model A.

.

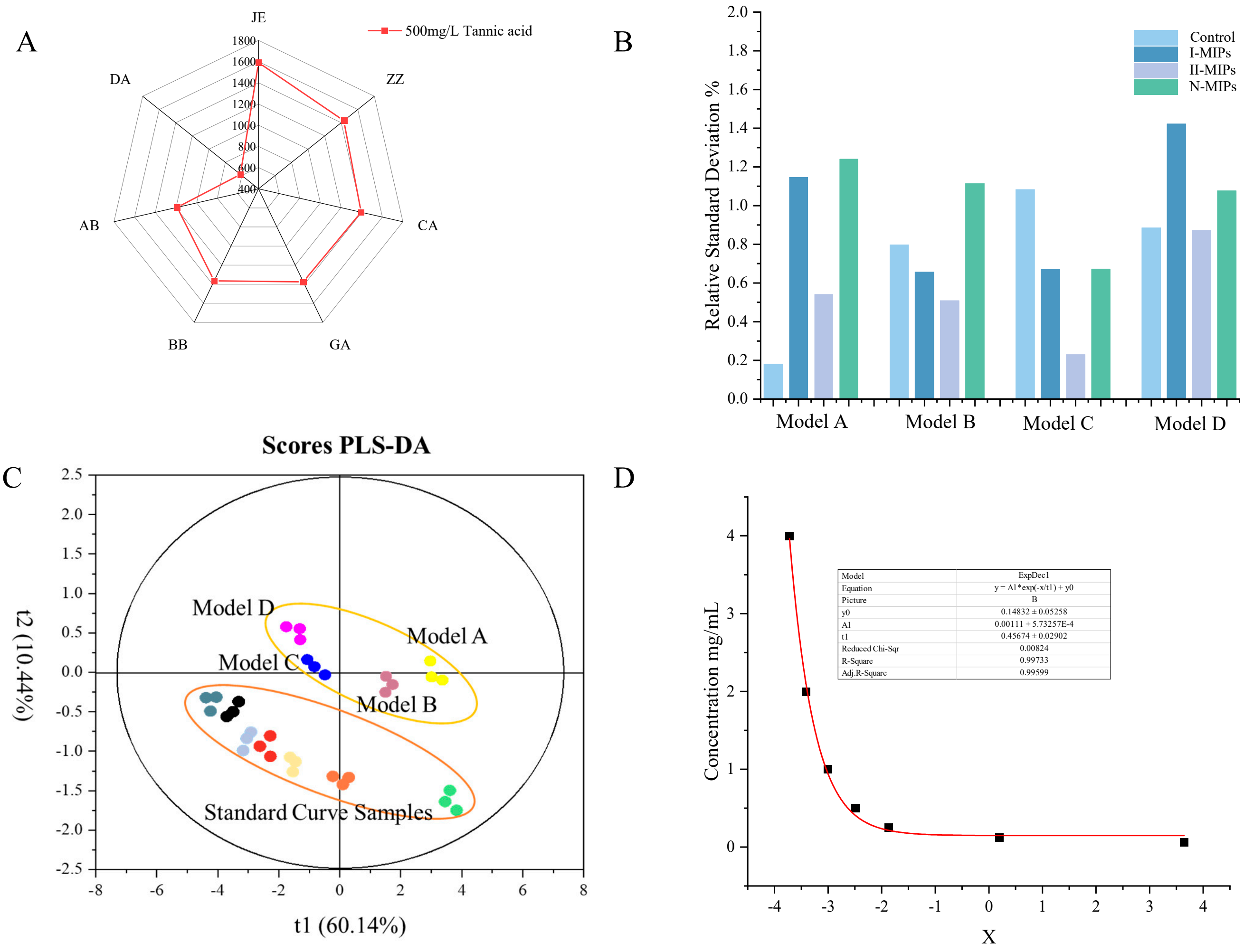

Figure S4. Electronic tongue quantification of astringency. A: Signal response (mV) of seven electronic tongue sensors to 500 mg/mL tannic acid; B: Relative standard deviation of samples between 40 and 120 s. Control represents untreated samples; C: Standard curve of tannic acid, and partial least square map of samples; D: Fitting curve between concentration of tannic acid (0.0625 mg/mL, 0.125 mg/mL, 0.25 mg/mL, 0.50 mg/mL, 1.0 mg/mL, 2.0 mg/mL, 4.0 mg/mL) and partial least square abscissa space.
